# Low level of polymorphism in two putative *NPR1 *homologs in the *Vitaceae *family

**DOI:** 10.1186/1745-6150-5-9

**Published:** 2010-02-05

**Authors:** Karine Bergeault, Christophe Bertsch, Didier Merdinoglu, Bernard Walter

**Affiliations:** 1 Laboratoire Vigne, Biotechnologies & Environnement, Université de Haute Alsace, 33 rue de Herrlisheim, 68000 Colmar, France; 2 Laboratoire de Génétique et Amélioration de la Vigne, INRA et Université de Strasbourg (UMR1131), 28 rue de Herrlisheim, 68000 Colmar, France

## Abstract

**Background:**

Grapevine is subjected to numerous pests and diseases resulting in the use of phytochemicals in large quantities. The will to decrease the use of phytochemicals leads to attempts to find alternative strategies, implying knowledge of defence mechanisms. Numerous studies have led to the identification of signalling pathways and regulatory elements involved in defence in various plant species. Nonexpressor of Pathogenesis Related 1 (NPR1) is an important regulatory component of systemic acquired resistance (SAR) in *Arabidopsis thaliana*.

**Results:**

Two putative homologs of *NPR1 *gene were found in the two sequenced grapevine genomes available in the Genoscope database for line 40024 and in the IASMA database for Pinot noir ENTAV 115. We named these two *NPR1 *genes of *Vitis vinifera *: *VvNPR1.1 *and *VvNPR1.2*. A PCR-based strategy with primers designed on exons was used to successfully amplify *NPR1 *gene fragments from different *Vitaceae *accessions. Sequence analyses show that *NPR1.1 *and *NPR1.2 *are highly conserved among the different accessions not only *V. vinifera *cultivars but also other species. We report nucleotide polymorphisms in *NPR1.1 *and *NPR1.2 *from fifteen accessions belonging to the *Vitaceae *family. The ratio of nonsynonymous to synonymous nucleotide substitutions determines the evolutionary pressures acting on the *Vitaceae NPR1 *genes. These genes appear to be experiencing purifying selection. In some of the species we have analysed one of the two alleles of *NPR1.1 *contains a premature stop codon. The deduced amino acid sequences share structural features with known NPR1-like proteins: ankyrin repeats, BTB/POZ domains, nuclear localization signature and cysteines. Phylogenetic analyses of deduced amino acid sequences show that VvNPR1.1 belongs to a first group of NPR1 proteins known as positive regulators of SAR and VvNPR1.2 belongs to a second group of NPR1 proteins whose principal members are AtNPR3 and AtNPR4 defined as negative regulators of SAR.

**Conclusion:**

Our study shows that *NPR1.1 *and *NPR1.2 *are highly conserved among different accessions in the *Vitaceae *family. VvNPR1.1 and VvNPR1.2 are phylogenetically closer to the group of positive or negative SAR regulators respectively.

**Reviewers:**

This article was reviewed by Fyodor Kondrashov, Purificación López-García and George V. Shpakovski.

## Background

The *Vitaceae *family consists of approximately 700 species classified in 14 genera. *Ampelopsis *and *Parthenocissus *are two genera related to each other. The genus *Vitis *has been divided into two distinct sections called *Euvitis *and *Muscadinia *[1]. These two sections can be differentiated not only on morphological and anatomical appearance but also on chromosome number. *Muscadinia *have 2n = 40 chromosomes while *Euvitis *have only 2n = 38 [2]. *Vitis vinifera*, as well as several other species and hybrids of *Vitis*, are well known economically for grapes, wine and raisins productions. Grape and their derivatives have an increasing worldwide market [3]. Vineyard is constantly subjected to numerous stresses due to climatic conditions, farming techniques, mineral deficiency or pathogens. Vineyards are threatened by numerous viruses, bacteria and fungi. This situation leads wine growers to use chemicals in large quantities.

The stimulation of grapevine's own defences could be an alternative strategy to phytochemicals. Numerous studies have led to the identification of several plant genes involved in defence mechanisms and especially in signal transduction pathways [4]. *Nonexpressor of PR 1 *(*NPR1*) gene of *Arabidopsis thaliana *is an important regulatory component of systemic acquired resistance (SAR) [5-7]. SAR is a plant immune response that is triggered after local infection with pathogens [8-10]. The onset of SAR requires the accumulation of salicylic acid (SA) and the coordinated expression of pathogenesis-related (PR) genes [7]. Besides SA-mediated SAR, other pathways are involved in plant defence systems, namely ISR (induced systemic resistance) and jasmonic acid (JA)-mediated pathways. The SAR and ISR pathways are independent but have an overlapping requirement for NPR1 [11]. When SA and JA are applied together to leaves, the presence of SA inhibits JA synthesis and signalling. This inhibition is alleviated in the *npr1 *mutant, indicating that NPR1 is part of the crosstalk control between signalling pathways [12]. NPR1 is a positive regulator of the plant defence response and its mechanism of action is well characterized.

The NPR1 protein localizes both to the cytoplasm and the nucleus [13]. Upon pathogen attack, accumulation of SA causes a decrease in cellular reduction potential, leading to the conversion of NPR1 from inactive oligomers into active monomers that enter the nucleus and interact differentially with TGA proteins which are basic-region leucine zipper (bZIP) transcription factors. This interaction stimulates the DNA binding activity of TGA factors to SA-responsive elements in the promoter of pathogenesis-related genes [8,14,15]. The *Arabidopsis thaliana *genome contains six *NPR1*-related genes [16] (called *AtNPR1 *to *AtNPR6*), whose deduced proteins all share the Broad Complex, Tramtrack and Bric a brac/Pox virus and Zinc finger (BTB/POZ) domain and the Ankyrin Repeat Domain (ARD). According to Mou *et al*. (2003) [15], AtNPR1 and NPR1-like proteins from four plant species contain ten conserved cysteines. Cys 82 and Cys 216 are essential for keeping NPR1 in the cytoplasm [15]. *NPR1 *genes have been found in genomes of various species such as rice [17,18], Indian mustard [19], apple [20] and cotton [21]. Overexpression of *AtNPR1 *in *Arabidopsis thaliana*, rice, tomato and wheat enhances fungal and bacterial resistance [17,20,22-27] through elevated expression of *PR *genes [22,25].

In plants, molecular diversity was first studied based on the existence of mutational events. Detection of single nucleotide polymorphisms (SNPs) allows the analysis of sequence differences between alleles. Nucleotide diversity reflects the combined history of selection, migration, recombination and mating systems experienced by species. Nucleotide diversity is one source of phenotypic variation [28]. SNPs have been characterized in crop plant genomes such as maize, sugarbeet, barley, soybean, wheat, rice and grapevine [29]. As the grapevine is propagated vegetatively, its genome has a higher probability to accumulate large deletions, insertions, inversions or other events which may differentiate the two pairs of chromosomes.

In the present study, a PCR-based strategy with primers designed on exons was used to successfully amplify *NPR1 *exons. A phylogenetic tree was used to analyse the relationship of NPR1 proteins from *Arabidopsis thaliana *and homologs from various plant species. Analysis of sequence mutations was done to study the polymorphism of *NPR1 *in fifteen accessions of the *Vitaceae *family. To examine the evolutionary pressures acting on the *NPR1.1 *and *NPR1.2*, the ratio of nonsynonymous (replacement) to synonymous (silent substitution) nucleotides was determined.

## Results and discussion

### Putative homologs of *AtNPR1 *in grapevine: *VvNPR1.1 *and *VvNPR1.2*

**Figure 1:**
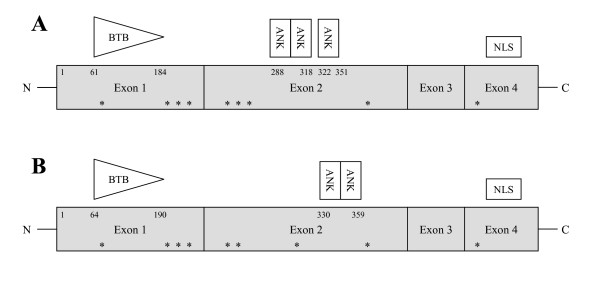
**Schematic representation of *VvNPR1.1 *(A) and *VvNPR1.2 *(B)**. The positions of the BTB/POZ, ankyrin repeat domains, nuclear localization signature and conserved cysteine residues represented by stars are shown. Numbers represent amino acid positions.

We named *VvNPR1.1 *and *VvNPR1.2 *two putative homologs of the *AtNPR1 *gene from the grapevine genome sequences published by the French-Italian Public Consortium for Grapevine Genome (line 40024) and by the Istituto Agrario Di San Michele All'Adige (*Vitis vinifera *Pinot noir ENTAV 115). Line 40024 [30] is highly homozygous and Pinot noir ENTAV 115 [31] is heterozygous. No additional member of the *NPR1 *gene family was found in NCBI and ESTAP databases after blast and key words research. The intron-exon structure in *NPR1.1 *and *NPR1.2 *from the *Vitaceae *accessions was the same as in *AtNPR1*: four exons and three introns, except for *Ampelopsis japonica *where intron 3 is missing. The complete cDNA is 1,755 bp for *VvNPR1.1 *and 1,764 bp for *VvNPR1.2*. The deduced proteins have 584 and 587 amino acids, respectively and they share structural features with known NPR1 proteins which are highly conserved across many species: ankyrin repeats in the middle of the protein (ARD), the N-terminal BTB/POZ domains, the C-terminal nuclear localization signature (NLS) [32] and nine among ten conserved cysteines described by Mou *et al*. (2003) [15] (Figure 1). This suggests that VvNPR1 may interact with other proteins, as previously described in *Arabidospsis thaliana *[13,33-36].

**Figure 2:**
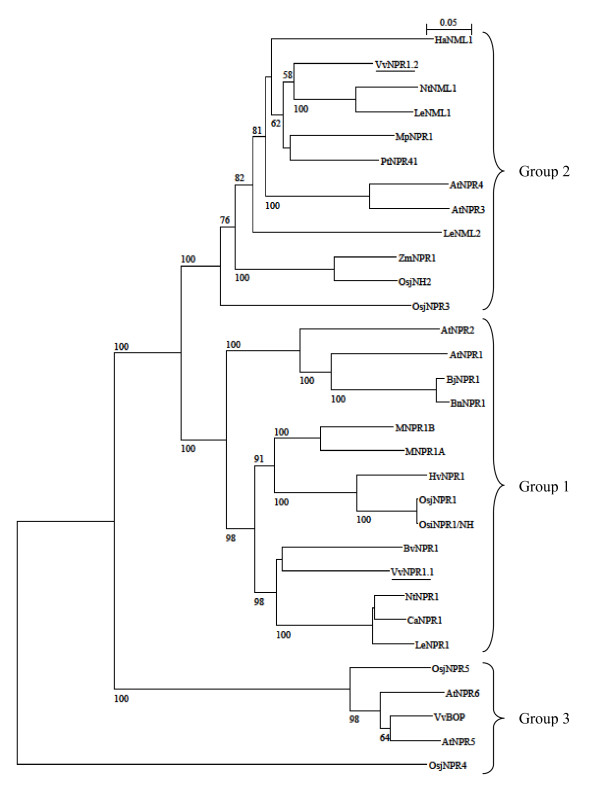
**Comparison of VvNPR1.1, VvNPR1.2 and putative Blade-on-petiole (VvBOP) with NPR proteins from *Arabidopsis thaliana *and other plant species**. Deduced sequences of VvNPR1.1 and VvNPR1.2 were compared to sequences of proteins annotated as being homologs of AtNPR1 from different plant species. Accession numbers used in the alignments are listed in table 1. All sequences annotated as *NPR1 *or closely related to *VvNPR1 *genes after blast were recovered in the NCBI database. The numbers beside the branches represent bootstrap values based on 5,000 replicates. The scale at the top indicates genetic distance proportional to the number of substitutions per site.

The amino acids 61-184 of VvNPR1.1 and 64-190 of VvNPR1.2 show similarity to the BTB/POZ motif. The amino acids 259-318 and 322-351 of VvNPR1.1 and amino acids 296-326 and 330-359 of VvNPR1.2 reveal respectively three and two highly conserved ankyrin repeats (Figure 1). The deduced amino acid sequences of VvNPR1.1 and VvNPR1.2 show 43% identity. VvNPR1.1 and VvNPR1.2, respectively, show 52% and 37% identity with AtNPR1. Three phylogenic groups have been described in the AtNPR1 protein family by Hepworth *et al*. (2005) [37] (Figure 2). These authors stated that branching pattern suggests that members in each group may have similar functions. VvNPR1.1 is close to AtNPR1 and AtNPR2 in the first group. VvNPR1.2 is with AtNPR3 and AtNPR4 in the second group. AtNPR5 and AtNPR6 present high homology with Blade-on-petiole 1 and 2 of *A. thaliana *(BOP1 and BOP2) which involved in some aspects of morphogenesis and leaf/flower development [37]. A putative *Blade-on-petiole *gene which we named *Vitis vinifera BOP *(*VvBOP*) was identified in the Genoscope database. VvBOP is closely related to AtNPR5 and AtNPR6 and can be involved in morphogenesis and leaf/flower development, like AtNPR5 and AtNPR6 (Figure 2) [6,16,19,20,23,30,38-40].

### Purifying selection for *Vitaceae NPR1.1 *and *Vitaceae NPR1.2*

**Figure 3:**
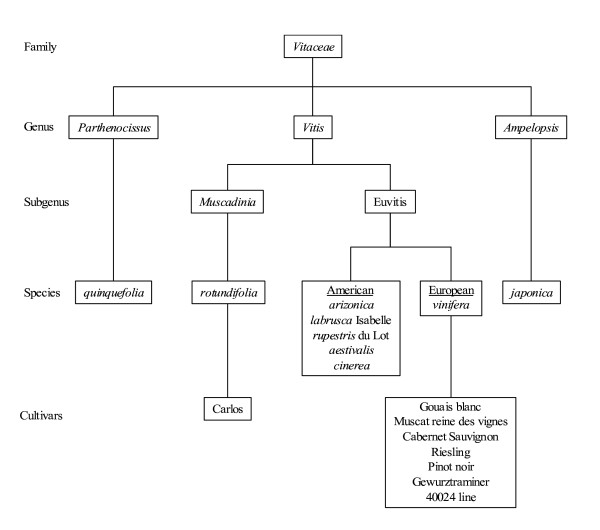
**Partial classification of *Vitaceae *[according to Gallet (1967) **[**41**]**]**.

**Figure 4:**
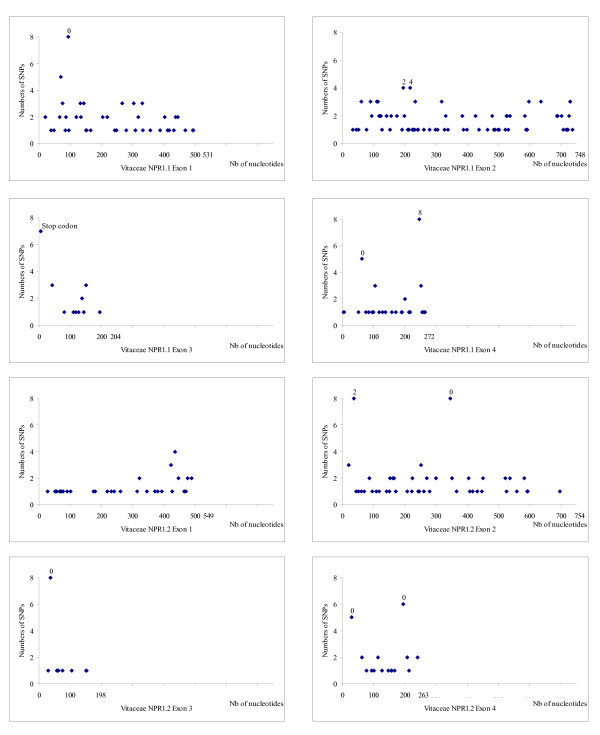
**Distribution of SNPs in *NPR1.1 *and *NPR1.2 *along exons 1 to 4**. From *Ampelopsis japonica*, *Muscadinia rotundifolia *Carlos, *Parthenocissus quinquefolia *and five American *Vitis *species. The horizontal scale indicates the nucleotide number. The vertical scale indicates the number of SNPs counted at each position among the eight species in comparison to line 40024. Numbers represent missense substitutions.

**Figure 5:**
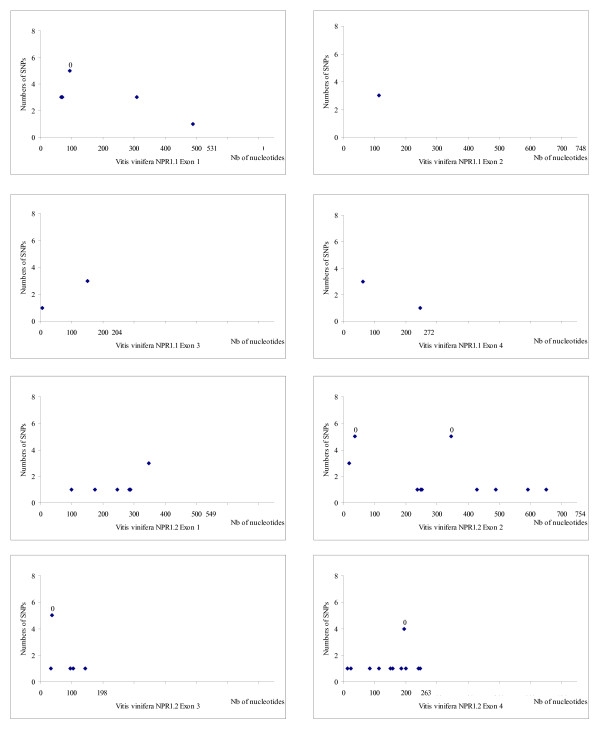
**Distribution of SNPs in *NPR1.1 *and *NPR1.2 *along exons 1 to 4**. From six *Vitis vinifera *cultivars (Gouais blanc, Muscat reine des vignes, Cabernet Sauvignon, Riesling, Pinot noir, Gewurztraminer). The horizontal scale indicates the nucleotide number. The vertical scale indicates the number of SNPs counted at each position among the eight species in comparison to line 40024. Numbers represent missense substitutions.

Sequence data for fifteen accessions belonging to the *Vitaceae *family (Figure 3) were obtained from *NPR1 *fragments amplified using PCR primers. Identification of polymorphic sites was based on two alleles sequenced. Figures 4 and 5 show the distribution of SNPs along the four exons of *NPR1.1 *and *NPR1.2 *respectively, in *Ampelopsis japonica*, *Muscadinia rotundifolia, Parthenocissus quinquefolia *and five American *Vitis *species and in six *V. vinifera *cultivars (Gouais blanc, Muscat reine des vignes, Cabernet Sauvignon, Riesling, Pinot noir, Gewurztraminer). The SNPs are not evenly distributed. *NPR1.1 *exon 3 and the four *NPR1.2 *exons have a low SNP rate (4% to 5.4% SNPs per exon), in comparison with *NPR1.1 *exons 1, 2 and 4 (7.2% to 8.4% SNPs per exon). Globally, *NPR1.1 *is more polymorphic than *NPR1.2*. In the six *V. vinifera *cultivars, SNP rate is lower than 2.5% (0.1% in *VvNPR1.1 *exon 2 to 2.5% in *VvNPR1.2 *exon 3) and 4.2% in *VvNPR1.2 *exon 4. In the six *V. vinifera *cultivars, SNP rate in introns (about 2%, data not shown) was not significantly different from SNP rate in exons [41].

#### *NPR1.1*

Polymorphisms in exon 1 and exon 4 of *NPR1.1 *were identified by silent substitutions at positions 23, 31, 515 and a missense substitution at position 576. A threonine codon present in all the other accessions changed in an asparagine codon in line 40024. Missense substitutions in the BTB/POZ domain cause protein modifications (Figure 1). It is likely that substitutions have important consequences for the function of the BTB/POZ domain and perhaps its regulation. In fact, the BTB/POZ domain is an evolutionarily conserved protein-protein interaction motif [42]. A mutation in this region results in the loss of NPR1 function and affects protein-protein interaction [6,7]. A heterozygous zone, observed in *Ampelopsis japonica*, *Muscadinia rotundifolia*, *Parthenocissus quinquefolia, V. cinerea*, *V. aestivalis*, *V. rupestris*, *V. arizonica *and *V. vinifera *Cabernet Sauvignon, at the beginning of *NPR1.1 *exon 3, shows a nucleotide substitution in a glycine codon (GGA) which introduces a premature stop codon (TGA) in the central part of the protein. These results suggest that one of the two *NPR1.1 *alleles generates a truncated protein (427 aa) derived from translation of only exon 1 and 2. As a result, the truncated protein does not retain one of the conserved cysteines, but contains all of the protein-protein interaction domains. The truncated protein VvNPR1.1 should be functional because it possesses the interaction domain and nine of ten conserved cysteines. The missing cysteine is not essential for the nuclear localization [15]. Nonsense substitutions that generate a premature translational termination signal should reduce the steady-state accumulation of the corresponding mRNA [43]. No readthrough mechanism have been described until now in plants and nonsense substitutions are not enough to stop the translation since the second allele can be entirely expressed. Alleles rendered non-functional due to mutations causing frame shifts and/or premature stop codons were observed for five defence response loci (EDS5, ESP, ETR1, EDS1 and PAD4) in *A. thaliana *[44]. There is no evidence for non-functional *AtNPR1 *alleles but it could be different for *VvNPR1.1*. The ankyrin repeat domain of AtNPR1 are necessary and sufficient for the interaction with members of the TGA family of transcription factors, although high-affinity interactions also require the N-terminal one third of NPR1 [13,35,36].

#### *NPR1.2*

A deletion of 3 amino acids (amino acids 17 to 19) and a supplementary pattern of 6 amino acids were found in exon 4 of *Ampelopsis japonica NPR1.2*. This supplementary pattern is not a preserved protein pattern according to web-based SMART program.

Polymorphisms in exons 2, 3 and 4 of *NPR1.2 *were identified by silent substitutions at positions 298, 446 and 565 and a change at position 195 from a leucine codon into a valine in *Ampelopsis japonica *and *Parthenocissus quinquefolia*.

According to Bakker *et al*. (2008) [44], there are three distinguished evolutionary scenarios for selection: (1) long-term balancing selection, where a gene may have highly diverged alleles at intermediate frequencies and a high level of silent polymorphism, (2) positive selection of a favorable allele, which can generate a locus with few, relatively young alleles with extended haplotypes and (3) purifying selection or functional constraint, which leads to low levels of nonsynonymous polymorphism and a commensurately low rate of divergence between species.

From our results, the ratio of nonsynonymous to synonymous nucleotide substitutions (Ka/Ks) relating influence of selection is smaller than 1 in both genes (0.84 in *NPR1.1 *and 0.44 in *NPR1.2*). These results suggest a purifying selection against substitutions that would result in amino acid replacements. In *NPR1.2*, the ratio is also lower than 1 for the four exons taken independently (0.34 to 0.68). The comparison of selected clones from fifteen accessions belonging to the *Vitaceae *family revealed a high homology for the two genes. The polymorphism data in *NPR1.1 *and *NPR1.2 *indicate purifying selection and sequence conservation. Generally, the defence response genes tend to maintain lower levels of diversity. The majority of defence response genes appear to be experiencing purifying selection [44]. In general, defence response genes do not appear to be under balancing selection but strong evidence of balancing selection was detected at *AtNPR1 *[44,45]. Our results show that *NPR1.1 *and *NPR1.2 *are highly conserved under strong purifying selection and do not vary much from accession to accession, indicating that mutations probably have deleterious consequences. Purifying selection results in the reduction of genetic variation through the elimination of maladjusted alleles and consequently of the mutations that caused the maladjustment. These contradictory results may be related to the fact that *A. thaliana *multiplies by sexual reproduction while grapevine by vegetative propagation.

### Functional hypothesis: two types of regulations by *NPR1*-like genes?

**Figure 6:**
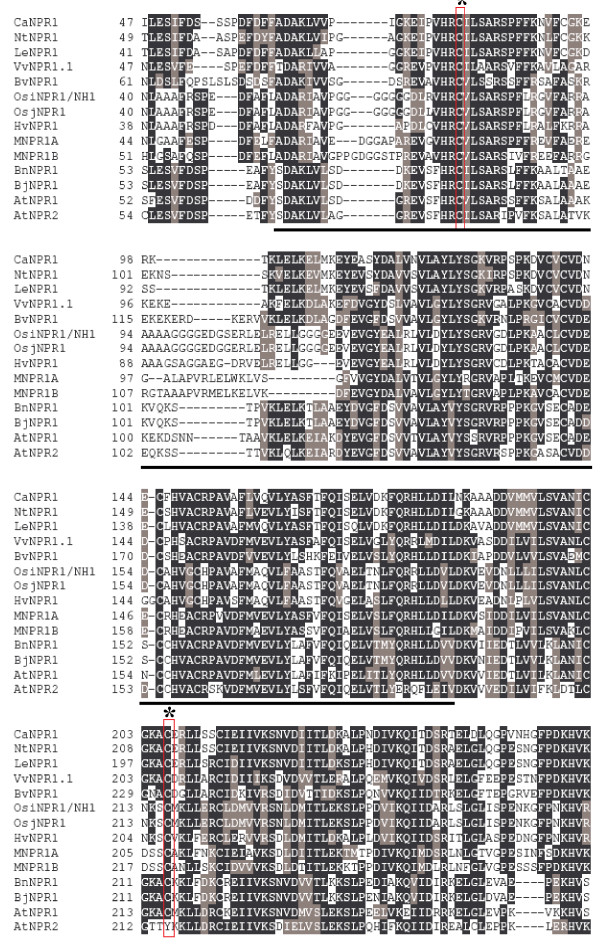
**Multiple alignment (clustal W) of *VvNPR1.1 *with *AtNPR1*, *AtNPR2 *and other *NPR1 *homologs**. Vertical rectangles and stars represent conserved cysteine residues. The BTB/POZ domain is underlined. Dashes indicate gaps introduced to maximize alignments.

The complete sequence of putative homologs of the *AtNPR1 *gene family is known in various species (Table 1). According to the phylogenetic tree (Figure 2), three groups of NPR genes can be distinguished. VvNPR1.1 is in the same group as NPR1 from sugar beet, tobacco, sweet pepper and tomato as also AtNPR1 and AtNPR2 from *Arabidopsis *and rice OsiNPR1/NH1. From the ten cysteines described as conserved in AtNPR1 and NPR1-like proteins, only eight are conserved in all the *Vitaceae *accessions we have analyzed, but the two cysteines essential for oligomer formation are present [15] (Figure 6). Sequence similarities, conservation of functional sites and cysteines suggest that VvNPR1.1 could have functions similar to that of AtNPR1 as a positive regulator of SAR. Nevertheless, functions of a limited number of NPR1 homologs have been studied. The expression of *BjNPR1*, *MNPR1A *and *MNPR1B *is induced by SA or MeJA treatment. BjNPR1-GFP fusion protein is localized to the nucleus following SA treatment. *MNPR1A *and *MNPR1B *increase PR gene expression [38]. Overexpression of *BnNPR1 *from canola and *OsiNPR1/NH1 *in rice complement the *npr1 *mutations and enhance resistance [23,46,47]. The NPR1 proteins in the first group could be activated during potential redox modifications induced by SA or various pathogenic conditions and show a similar regulation across different species.

**Table 1:** Proteins from the NPR1 family in different plants species

Taxon	GenBank Accession Numbers	References
Dicots		
*Arabidopsis thaliana (AtNPR1)*	AT1G64280	Cao *et al*., 1997 [6]
*Arabidopsis thaliana NPR2 (AtNPR2)*	NM_118745	Liu *et al*., 2005 [16]
*Arabidopsis thaliana NPR3 (AtNPR3)*	NM_123879	
*Arabidopsis thaliana NPR4 (AtNPR4)*	NM_118086	
*Arabidopsis thaliana NPR5 (AtNPR5)*	NM_129700	
*Arabidopsis thaliana NPR6 (AtNPR1)*	NM_115572	
*Beta vulgaris (BvNPR1)*	AY640381	Meur *et al*., 2006 [19]
*Brassica juncea (BjNPR1)*	AY667498	
*Brassica napus (BnNPR1)*	AF527176	
*Capsicum annuum (CaNPR1)*	ABG38308	
*Helianthus annuus NIM1-like protein 1 (HaNML1)*	AY640383	
*Hordeum vulgare (HvNPR1)*	AM050559	
*Lycopersicon esculentum (LeNPR1)*	AY640378	
*Lycopersicon esculentum NIM1-like protein 1 (LeNML1)*	AY640379	
*Lycopersicon esculentum NIM1-like protein 2 (LeNML2)*	AY640380	
*Malus × domestica cultivar (MpNPR1)*	EU624123	Malnoy *et al*., 2007 [20]
*Musa acuminata (MNPR1A)*	DQ925843	Endah *et al*., 2008 [38]
*Musa acuminata (MNPR1B)*	EF137717	Zwicker *et al*., 2007 [39]
*Nicotiana tabacum (NtNPR1)*	DQ837218	Jaillon *et al*., 2007 [30]
*Nicotiana tabacum NIM1-like protein 1 (NtNML1)*	AY640382	Jaillon *et al*., 2007 [30] Jaillon *et al*., 2007 [30]
*Populus trichocarpa (PtNPR41)*	DQ481233	
*Vitis vinifera NPR1.1 (VvNPR1.1)*	CAO65332	
*Vitis vinifera NPR1.2 (VvNPR1.2)*	CAN67078	
*Vitis vinifera BOP (VvBOP)*	CAO23333	
		

Monocots		
*Oryza sativa indica (OsiNPR1/NH1)*		Chern *et al*., 2005 [23]
*Oryza sativa japonica (OsjNPR1)*		Ohyanagi *et al*., 2006 [40]
*Oryza sativa japonica (OsjNH2)*		
*Oryza sativa japonica (OsjNPR3)*		
*Oryza sativa japonica (OsjNPR4)*		
*Oryza sativa japonica (OsjNPR5)*		
*Zea mays (ZmNPR1)*		

**Figure 7:**
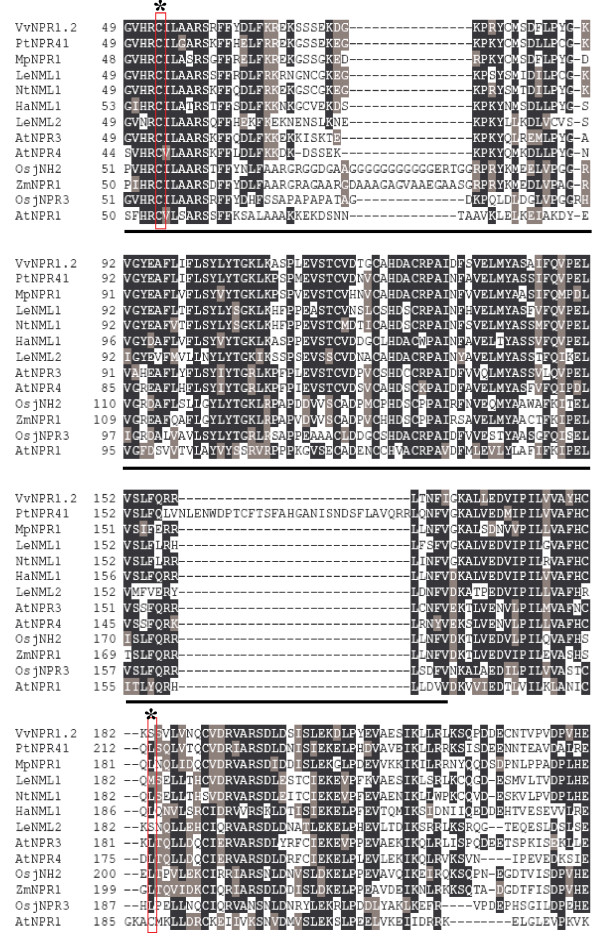
**Multiple alignment (Clustal W) of *VvNPR1.2 *with *AtNPR1*, *AtNPR3*, *AtNPR4 *and other *NPR1 *homologs**. Vertical rectangles and stars represent conserved cysteine residues. The BTB/POZ domain is underlined. Dashes indicate gaps introduced to maximize alignments.

VvNPR1.2 is grouped with NtNML1 and LeNML1 as also AtNPR3 and AtNPR4 which have been described as negative regulators of SAR (Figure 2) [34]. Only five of the ten cysteine residues are conserved in the proteins from the group 2. Cysteine 216 which is essential for oligomerization in AtNPR1 is absent from all the NPR1 in group 2. The absence of cysteine residue 216 (Figure 7) suggests that VvNPR1.2 should be differently regulated, as observed for AtNPR3 and AtNPR4. AtNPR3 and AtNPR4 perform overlapping functions and that loss of the function in *npr3 npr4 *double mutant leads to much higher *PR-1 *expression and enhanced resistance [34]. It is plausible that AtNPR3 and AtNPR4 negatively regulate *PR-1 *expression and pathogen resistance [34]. Inactivating both AtNPR3 and AtNPR4 leads to activation of TGA and expression of *PR *genes [34]. AtNPR3 and AtNPR4 would be negative regulators of plant defence responses [34]. A contradictory result has been reported with *MpNPR1-1 *which is induced by the SAR and its overexpression increases the resistance of apple to pathogens, suggesting that MpNPR1 may act as a positive regulator despite the absence of cysteine 216 [20]. Therefore, phylogenetic analysis is not sufficient to predict a positive or negative control of defence responses for *VvNPR1.2*.

## Conclusions

Homologs of the Arabidopsis *NPR1 *gene have now been isolated from numerous other plant species. Our study with Genoscope annotations provides the existence of a possible *NPR1 *gene family in *Vitaceae*. *VvNPR1.1 *and *VvNPR1.2 *genes have four exons and three introns. The deduced amino acid sequences show 52% and 37% identity to AtNPR1, and all three functional domains identified in *A. thaliana *NPR1 are conserved in the *Vitaceae NPR1*. The polymorphism on these two genes is in favour of purifying selection. Two *Vitis vinifera *NPR1-like proteins are separated in two of the three groups described by Hepworth *et al*. (2005) [37]. We have shown that the VvNPR1.1 is related to a first NPR1 group of positive regulators of SAR and VvNPR1.2 is related to a second NPR1 group whose principal members would be negative regulators of SAR, AtNPR3 and AtNPR4.

## Methods

### Plant material and DNA extraction

*Ampelopsis japonica*, *Muscadinia rotundifolia *cv. Carlos, *Parthenocissus quinquefolia*, five American *Vitis *species (*arizonica, labrusca *Isabelle, *rupestris *du Lot, *aestivalis *and *cinerea*), six *Vitis vinifera *cultivars (Gouais blanc, Muscat reine des vignes, Cabernet Sauvignon clone 169, Riesling clone 49, Pinot noir clone 162, Gewurztraminer clone 46) and *V. vinifera *inbred line 40024 [48] [derived from Pinot noir, and bred close to full homozygosity (estimated at about 93%) by successive selfings [30] were used. The reference sequence used for SNP analysis was from line 40024 [48]. Classification of the various accessions used in this study is schematized in figure 3.

40024 genomic DNA was extracted using DNeasy^® ^Plant Mini Kit (Qiagen, Courtaboeuf, France) following the manufacturer's recommendations and the other DNAs were a generous gift from Dr F. Pelsy (INRA, Colmar, France).

### Amplification of DNA

PCR primers to amplify exons using the published genome sequence http://www.cns.fr/externe/English/corps_anglais.html[30] were designed (Table 2). Genomic DNA was amplified by PCR using the following conditions: 10 ng of DNA template, 1 × PCR Buffer (Invitrogen), 1.5 mM MgCl_2_, 200 μM each dNTP (Invitrogen), 0.2 μM each primer, 1.25 Unit Platinum^® ^Taq DNA Polymerase (Invitrogen) and milli-Q^® ^water to a final volume of 25 μl. PCR reactions were performed using a 5 min initial denaturation/activation step, followed by 35 or 40 cycles at 94°C for 45 s, Tm for 1 min, and 72°C for 1 min per Kb, with a final extension step of 10 min at 72°C. PCR products were assessed by electrophoresis in 1.5% agarose gels and visualized by ethidium bromide staining.

**Table 2:** Sequence of primer derived from line 40024 (Genoscope Vitis Genome Browser) to amplify the exons of *VvNPR1.1 *and *VvNPR1.2*

Gene	Forward primers (5'->3')	Reverse primers (5'->3')	Amplified size (bp)
VvNPR1.1	ATGGACTACAGAGCTGCTC	CTGATAAAGGCCGACCAAT	531
	AGACGCCTGATGGACATTC	CTCTATTTTCCTTGAGGTACAACAG	748
	TTGGACTAGCAAAACTTCTATTCC	CAGTTTTGGACAGTGCTCTTAGCC	204
	TGGACCTTGGGAAACGCTTTT	TTACTTCTTGCAAGAGAGTCTA	272

VvNPR1.2	ATGGCCAATTCAGCTGAGC	CTGAAAAAGTGAGACCAGCTCTGGTA	549
	CGGCGTCTTACCAACT	CTCTATTTTCCAGGTACAG	754
	TGGCATTTGCACGATTATTCTTCC	CTGTTTTCACAAGGGCATTCATCCTTGAACG	198
	TGGAGATGGGTCGACGC	TCATAATTTTCTAGCCTTGTGAC	263

### Cloning gene-specific PCR fragments

When polymorphism appeared after the first sequencing reaction, the PCR products were ligated into the pCR2.1 vector (Original TA Cloning Kit, Invitrogen) according to the manufacturer's instructions. Ligation mixtures were incubated overnight at 14°C to obtain the maximum number of transformants. Ligation products were transformed into competent *E.coli *bacteria cells (InαF') using a 90 second heat-shock at 42°C. The bacterial cells were spread onto LB agar plates containing ampicillin (100 μg/ml) (Sigma) and X-Gal (40 μg/ml) (Euromedex).

Plasmid minipreps were performed by the alkaline lysis method [49]. The PCR fragments in the vectors were sequenced by Genoscreen (Lille, France) using M13 and M13 rev primers.

### Polymorphism detection methods

A PCR-based strategy with primers designed on exons amplifying successfully *NPR1 *gene fragments was used. The sequencing reaction can produce one (homozygote) or two (heterozygote) peaks depending on the genotype for each nucleotide. DNA sequences and the complete protein sequences of members of the *NPR1 *gene family were aligned using Vector NTI and Bioedit software. Phylogenetic trees were produced using the ClustalW web site http://align.genome.jp/ and the Phylo_win program using the neighbour-joining method [50]. The *NPR1 *sequences (Table 1) from other species, available in the NCBI GenBank http://www.ncbi.nlm.nih.gov/, were included in the sequence analysis for comparison. The Web-based SMART program was used to predict the BTB/POZ domains and to confirm the localization of ankyrin repeats for each protein. Percentage of SNP corresponds to the ratio of number of modified nucleotides to total number of nucleotides for each exon. To examine the evolutionary pressures acting on the *NPR1.1 *and *NPR1.2 *genes, the ratio of nonsynonymous (replacement) substitutions to synonymous (silent) nucleotide substitutions was determined [51-53]. Generally, values larger than 1 indicate sequence diversification. Instead, values smaller than or equal to 1 are indicative, respectively, of sequence conservation or random mutagenesis.

### Additional bioinformatics databases

The ESTAP software from Expressed Sequence Tag (EST) projects http://staff.vbi.vt.edu/estap/ was also used to identify *NPR1 *sequences. Phylogenetic trees were produced using the ClustalW Web site http://www.ebi.ac.uk/Tools/clustalw/index.html. Predicted amino acid sequences were generated using the DNA sequence translate tools EXPASY http://www.expasy.ch/tools/dna.html.

## Abbreviations

NPR1: non expressor of PR 1; SA: salicylic acid; JA: jasmonic acid; PR: pathogenesis-related; SAR: systemic acquired resistance; ISR: induced systemic resistance; BTB/POZ: broad complex, tramtrack and bric a brac/pox virus and zinc finger; ARD: ankyrin repeat domain; NLS: nuclear localization signal.

## Competing interests

The authors declare that they have no competing interests.

## Authors' contributions

KB carried out all the experiments, alignment and comparison of the sequences, phylogenetic analyses and drafted the manuscript. CB and BW initiated and conceived the study, have done general supervision and coordination and corrected the manuscript. DM participated in the design of the study and helped to organize the manuscript. All authors read and approved the final manuscript.

## Reviewers' reports

### Reviewer 1: Fyodor Kondrashov, Centre for Genomic Regulation, Barcelona, Spain

This manuscript suffers from conceptual flaws that renders it incomprehensible and misleading: the difference between mutations, polymorphisms and substitutions is obscured to a degree that I believe in the current form makes the analysis almost meaningless.

The authors determine the sequence of a duplicated orthologs of a NPR1 gene found in *Arhabidopsis *in several different species and strains of the *Vitaceae *family. The differences between the obtained sequences the authors interchangeably call "polymorphisms", "substitutions", "modifications" and "mutations". I can only assume that the authors have sequenced several individuals from each species or strain, since the title refers to polymorphisms. However, I did not find a statement of how many individuals were sequenced for each species/strain, or whether or not substitutions where included in the analysis. The authors state that "The NPR1 sequences (Table 1) from other species, [...], were included in the sequence analysis for comparison." What kind of comparison? Is the data really a mix of polymorphisms and substitutions? Such constant confusion between mutational, polymorphism and marco-evolutionary levels provides a huge barrier to comprehension of the results.

Another example: that authors claim that "[Ka/Ks] values smaller than or equal to 1 are indicative, respectively, of sequence conservation or random mutagenesis." Actually, Ka/Ks of any value can be achieved by random mutagenesis! Unfortunately, there are other, equally frustrating, examples. Before the authors can make a clear distinction between mutations (instantaneous products of mutagenesis), polymorphisms (segregating alleles in a population) and substitutions (sequence differences between homologs in different species or strains) and make it clear to the reader what kind of data they are using the manuscript will remain fatally flawed.

In addition, once the authors can distinguish between these three concepts the data analysis can be expanded to include a combination of phylogenetic and population genetic analyses, broadening the overall ability of the authors to infer the kinds of selection acting in these genes.

A minor issue that arises from the same misconception. The authors spend some time discussing the possible functional implications of different nonsynonymous and nonsense "mutations". All of these functional implications are a mute point if these are deleterious segregating polymorphisms. The functional implications are justified if the authors mean substitutions.

Author's response: *The manuscript was improved in response to these observations in particular to make it more comprehensible*.

*Pages 11 and 12, words "mutations" and "polymorphism" were used by Bakker et al. (2008)*.

*In the last paragraph before "Functional hypothesis *[...]*", the term "mutations" was used because it is a general conclusion*.

*We have sequenced a pool of three individuals for each species. Each PCR products was sequenced once. When polymorphism appeared after the first sequencing reaction, the PCR products were ligated into a vector and sequenced six times to generate the two polymorphic alleles, separately. We estimated that this pool is sufficient to represent the whole species studied because grapevine is multiplicated by vegetative propagation. Moreover, our reference 40024 line was also sequenced according to the same procedure, compared to the sequence available on the Genoscope database and no difference was observed. If an allele was identical to our reference but the other one was different, we took account to the polymorphic allele to consider a maximum diversity*.

*The sentence "The NPR1 sequences (Table *1) *from other species *[...], *were included in the sequence analysis for comparison." refers to Figure *2. *The aim of this study was to place our two genes in a set of gene described as NPR1. We used the raw sequence of 40024 line available on the Genoscope database to make our comparison without introducing polymorphisms or substitutions*.

*We have reduced the functional hypothesis part*.

Second review of Bergeault *et al*. "Low level of polymorphism in two putative orthologs of NPR1 genes in the Viraceae family".

While significantly improved from the last version, I still think that the manuscript suffers from conceptual inconsistencies. Also, now that the results are clearer to me I do not find them interesting or revealing.

The content: The authors show that two paralogs that have a 43% identity are under negative selection by comparing the density of nonsynonymous and nonsynonymous polymorphisms. This is hardly surprising and I am not sure why the authors think that such a result should be published. Any theory of gene duplications will confirm that nothing but negative selection is expected for such distant paralogs.

The approach: On the other hand, the authors have the right approach to study the functional impact of the functional impact of the origin of gene duplication, which is one of the least understood part of gene duplication evolution. If the paralogs were 99% identical than the results would have shown that the origin of a new function occurred quickly, or that the gene duplication itself was adaptive. Many studies of such sort should be undertaken, but this analysis does not mean very much with distant paralogs.

The language: Another unfortunate aspect of this manuscript is that it is littered with terminological inconsistencies. For example the authors say "To examine the evolutionary pressures acting on the *NPR1.1 *and *NPR1.2*, the ratio of nonsynonymous (replacement) to synonymous (silent substitution) nucleotides was determined." Clearly, the word "nucleotides" should be either "substitutions" or "polymorphisms". Another such misconception is the claim that Kn/Ks < 1 can signify a recent selective sweep. I disagree: the only other thing it can signify besides negative selection is different mutation rates of synonymous and nonsynoymous sites. Even more unfortunately, is that the authors really mean Pn/Ps (for polymorphisms, not substitutions) throughout the manuscript and use "substitutions" (which are fixed differences) instead of "polymorphisms" (which are still segregating in the population). Also, instead of using "accessions" some other term, for example "strain" should be used: one cannot extract DNA from an accession.

Author's response: *The manuscript was improved in response to these observations in particular to "recent selective sweep" where we are agreeing with Dr Kondrashov. This sentence was suppressed*.

*The "accession" is frequently used in wine-growing. This term indicates a grape variety, a variety or a clone*.

### Reviewer 2: Purificación López-García, Université Paris-Sud, Paris, France

Review of the article "Low level of polymorphism in two putative orthologs of NPR1 genes in the Vitaceae family by Bergeault *et al*., submitted to Biology Direct. Bergeault *et al*. identify two homologues of *Arabidopsis NPR1 *genes, known to be involved in general systemic resistance against pathogens or herbivores, in the genome of the grapevine *Vitis vinifera*. They subsequently design specific primers and amplify the corresponding genes from various grapevine cultivars. The analysis of those genes show a low level of polymorphism and, based on a ratio of synonymous versus non-synonymous substitutions lower than 1, the authors conclude that these genes are under purifying selection. Overall, the work is clear and appears well conducted. However, I think that the structure and writing of the manuscript may be improved. A few suggestions follow.

In particular, there are many figures, some of which could be eliminated without loss of essential information. This is the case of figure 6 showing the stop codon region in NPR1.1. As an additional suggestion, figure 1 and 4 could be merged together in a composite figure showing as an inset the NPR1.1 BTB/POZ domain region with the polymorphisms identified. I also believe that figure 8, showing the different cultivars analyzed, should appear as figure 1 at the beginning of the results section, since these are the cultivars subsequent work is about. Otherwise, placing it at the end of the manuscript with only a brief mention in the Methods section would make it dispensable.

The long discussion about the potential functions of NPR1 genes in *V. vinifera *is rather speculative in the absence of actual functional data and could be significantly shortened.

Bergeault et al. observed that "in six *V. vinifera *cultivars SNP rate to introns was not significantly different to exons". Why? Could this suggest that the low SNP observed is not (at least totally) due to purifying selection but to other kind of constraint acting on that particular genomic region?

Minor comments:

- First paragraph of Background. Some references should be added. Readers may be not familiar with *V. vinifera *and their general genomic characteristics.

- Page 12, last line before "Functional hypothesis...". Sexual instead of sexuel.

- Page 15, "high homology values". This expression is incorrect. Homology is an all-or-nothing quality. Either two genes are homologous (derive from a common ancestor) or they are not. "High similarity or identity values" would be more appropriate.

Author's response: *The manuscript was improved in response to these very useful comments. In answer to the question concerning the SNP rate to introns, it is not significantly different to exons and this for the 3 introns which compose each gene. We suppose indeed that it is due to purifying selection since it is observed in 3 introns in six *V. vinifera.

*For the incorrect expression in the minor comments, this sentence was suppressed*.

### Reviewer 3: George V. Shpakovski Shemyakin-Ovchinnikov Institute of Bioorganic Chemistry, Russian Academy of Sciences, Moscow, Russia

This is my second review of this manuscript (my previous comments were addressed by the authors in their new manuscript submission). The authors report the results of an experimental (PCR-based) and computational study of *NPR1 *[Nonexpressor of Pathogenesis Related 1]-like genes' polymorphism in different species of the *Vitaceae *family. Grapevine is subjected to numerous stresses (both enviromental and pathogeneous), and new knowledge on its defence mechanisms (*NPR1 *gene is one of the main component of systemic acquired resistance [SAR] in plants) certainly will be beneficial both for scientific community and for human society in general. The main finding of the manuscript is the fact that family of *NPR *genes in *Vitis vinifera *and probably in all other plants consists of at least 3 separate branches. These 3 subfamilies of *NPR *genes in plants exemplified in *Arabidopsis thaliana *by *AtNPR1*, *AtNPR3 *and *AtNPR5 *(*BOP*) genes, respectively. Analysis of nucleotide polymorphisms in two *NPR1-*like genes from fifteen accessions belonging to the *Vitaceae *family indicate that the genes are under purifying selection, but the *AtNPR1 *ortholog (*VvNRP1.1*) is more polymorphic. A tentative hypothesis of authors about two types (positive and negative) of regulation by different subfamilies of *NPR1*-genes although is interesting, but certainly needs some further experimental support. The revised version of the manuscript takes the suggestions that I previously made into account. The changes in the revised manuscript and the new title help to clarify the aim of the manuscript considerably. In my opinion, it would be better to re-name *Vitis *genes described by the authors on *VvNRP1*, *VvNRP3 *and *VvNRP5 *instead of *VvNRP1.1*, *VvNRP1*.2 and *VvBOP *at it is in the text. The manuscript might need some additional editing and proofreading.

Author's response: *We are grateful to Dr Shpakovski for his comments and notice very judicious. Indeed, the hypothesis about their functions is essentially based on a work on literature. But, we think that it was interesting to discuss the two types of SAR regulation. Moreover, we indicate in the manuscript "Therefore, phylogenetic analysis is not sufficient to predict a positive or negative control of defence responses for *VvNPR1.2."

*With regard to re-name *Vitis *genes, another classification would make confusion. For example, where are *VvNPR2 *and *VvNPR4*? Moreover, we have at present no data concerning *VvNPR1.2 *which would act as a negative regulator of SAR and it would be premature to re-name it*, VvNPR3.
